# Chemical inducer of regucalcin attenuates lipopolysaccharide‐induced inflammatory responses in pancreatic MIN6 β‐cells and RAW264.7 macrophages

**DOI:** 10.1002/2211-5463.13321

**Published:** 2021-11-09

**Authors:** Tomiyasu Murata, Kazunori Hashimoto, Susumu Kohno, Chiaki Takahashi, Masayoshi Yamaguchi, Chihiro Ito, Itoigawa Masataka, Roji Kojima, Kiyomi Hikita, Norio Kaneda

**Affiliations:** ^1^ Laboratory of Molecular Biology Faculty of Pharmacy Meijo University Nagoya Japan; ^2^ Division of Oncology and Molecular Biology Cancer Research Institute Kanazawa University Japan; ^3^ Cancer Biology Program University of Hawaii Cancer Center University of Hawaii at Manoa Honolulu HI USA; ^4^ Laboratory of Natural Products Chemistry Faculty of Pharmacy Meijo University Nagoya Japan; ^5^ School of Sport and Health Science Tokai Gakuen University Miyoshi Japan; ^6^ Laboratory of Analytical Pharmacy Faculty of Pharmacy Meijo University Nagoya Japan; ^7^ Department of Pharmacy Faculty of Pharmacy Gifu University of Medical Science Kani Japan

**Keywords:** derrisfolin A, inflammation, lipopolysaccharide, MIN6 cells, RAW264.7 macrophages, regucalcin

## Abstract

We previously isolated derrisfolin A, a novel rotenoid derivative, from the stems of *Derris* *trifoliata* Lour. (Leguminosae). Here, we report that derrisfolin A induces the expression of endogenous regucalcin (RGN) protein in both pancreatic MIN6 β**‐**cells and RAW264.7 macrophages. Induction of RGN expression by derrisfolin A or retrovirus‐mediated gene transfer in MIN6 cells and RAW264.7 macrophages significantly decreased lipopolysaccharide (LPS)‐induced mRNA expression of *Nos2*, *Il1b*, and *Tnf* via nuclear factor‐κB activation; reduced LPS‐induced apoptosis in MIN6 cells, accompanied by decreased production of nitric oxide, interleukin‐1β, and tumor necrosis factor‐α; and attenuated generation of LPS‐induced reactive oxygen species, malondialdehyde, and 3‐nitrotyrosine in MIN6 cells. Additionally, in co‐cultures of MIN6 cells with RAW264.7 macrophages in the presence of LPS, induction of RGN expression by derrisfolin A or retrovirus‐mediated gene transfer in RAW264.7 macrophages attenuated apoptosis and oxidative/nitrosative stress in MIN6 cells. These results suggest that the induction of RGN expression in MIN6 cells was effective in suppressing LPS‐induced inflammatory cytotoxicity and that in co‐culture conditions, the induction of RGN expression in RAW264.7 macrophages blocked LPS‐induced paracrine effects of RAW264.7 macrophages on inflammatory cytotoxicity in MIN6 cells. Our findings suggest that derrisfolin A, a chemical inducer of RGN, might be useful for developing a new drug against macrophage‐associated β‐cell inflammation in type 2 diabetes.

## Graphical abstract

Derrisfolin A, a chemical inducer of regucalcin, attenuated lipopolysaccharide‐induced inflammatory responses in pancreatic MIN6 β‐cells. Regucalcin in MIN6 cells and RAW264.7 macrophages also blocks inflammatory interaction between both cells in a transwell coculture system. Regucalcin might act as a suppressor of macrophage‐associated pancreatic β‐cell inflammation.

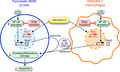

**Abbreviations**
3‐NT: 3‐nitrotyrosine
DMEM: Dulbecco’s modified Eagle’s medium
ECL: enhanced chemiluminescence
IL‐1β: interleukin‐1β
LPS: lipopolysaccharide
MAD: malondialdehyde
NF‐*κ*B: nuclear factor‐*κ*B
NF‐κB: nuclear factor‐κB
NO: nitric oxide
ROS: reactive oxygen species
sgRNA: single‐guide RNA
T2D: type 2 diabetes
TNF‐α: tumor necrosis factor‐α
TUNEL: terminal deoxynucleotidyl transferase biotin‐dUTP nick end labeling

Regucalcin (RGN) plays multiple regulatory roles in maintaining cell functions such as the cell defense mechanism. Several *in vitro* studies have demonstrated that RGN exerts a protective effect against apoptosis induced by various stimuli including lipopolysaccharide (LPS), tumor necrosis factor‐α (TNF‐α), and transforming growth factor‐β (TGF‐β) [1]. We previously reported that RGN also attenuated amyloid‐β‐induced apoptosis in neuronally differentiated PC12 cells *in vitro* [2]. In an *in vivo* study, transgenic overexpression of RGN in the prostate of rats improved age‐induced antioxidant defenses and prevented resistance to apoptosis [3]. Additionally, we reported that RGN acts as a suppressor protein in the proliferation of various human cancer cells by regulating intracellular signaling pathways [4, 5, 6, 7, 8, 9]. Furthermore, we recently discovered that RGN enhances the differentiation of 3T3‐L1 cells into adipocytes, which is accompanied by the upregulation of adipogenic differentiation markers [10].

We previously reported that hepatocyte RGN was upregulated by insulin and contributed to liver lipid metabolism via insulin action [11]. We also examined lipid metabolism in RGN transgenic rats and demonstrated that RGN overexpression induced increased levels of triglyceride, total cholesterol, and free fatty acids [11, 12]. *In vivo* RGN knockout (KO) mouse studies revealed that RGN is related to the pathogenesis of diabetic nephropathy, nonalcoholic fatty liver disease, and hepatic steatosis [13, 14]. Furthermore, impaired insulin secretion in RGN‐KO mice resulted in an abnormality of glucose tolerance [15, 16]. *In vitro* studies using pancreatic islets of RGN‐KO mice demonstrated that RGN deficiency caused impaired insulin secretion in response to glucose or potassium chloride, suggesting the involvement of RGN in insulin secretion in pancreatic β‐cells [15, 17].

Type 2 diabetes (T2D) is characterized by deficient insulin secretion from pancreatic β‐cells and insulin resistance in peripheral tissues [18]. Metabolic disorder‐triggered chronic inflammation plays an important role in the onset and development of T2D [19, 20]. In humans, T2D has been proposed as linked to compositional changes in the intestinal microbiota [21, 22]. Additionally, the serum concentration of LPS in diabetic patients is higher than that in healthy controls, indicating that the level of LPS is a risk factor in T2D development [23]. LPS induces proinflammatory mediators such as nitric oxide (NO), interleukin‐1β (IL‐1β), and TNF‐α in pancreatic β cells by activating the nuclear factor‐κB (NF‐κB) inflammatory pathway [24, 25, 26] and then impair β‐cell function [26, 27], leading to decreased insulin secretion potential. Thus, circulating LPS directly attacks β‐cells and triggers β‐cell inflammation and dysfunction [21, 22, 23], suggesting that strategies for reducing LPS‐induced β‐cell inflammation are useful to treat T2D patients.

Macrophages infiltrate the pancreatic islets in T2D patients and evoke islet inflammation, resulting in β‐cell dysfunction [28, 29, 30]. Evidence of crosstalk between macrophages and β‐cells via proinflammatory cytokines and chemokines in the islet microenvironment has been reported [29, 30]. For example, an elevated level of circulating free fatty acids causes macrophage‐mediated islet inflammation, leading to β‐cell dysfunction and apoptosis [31]. Furthermore, the proinflammatory cytokines secreted from macrophages contribute to β‐cell dysfunction in T2D [32]. Thus, blocking macrophage‐mediated β‐cell inflammation would be beneficial for preventing β‐cell dysfunction in T2D patients.

Numerous bioactive compounds have been identified from *Derris* *trifoliat*a Lour. [33, 34, 35, 36, 37]. We previously isolated some rotenoid compounds from *D*. *trifoliata* Lour. stems [38]. Further, we discovered the suppressive effect of derrisfolin A, a new rotenoid derivative, against LPS‐induced NO production in LPS‐stimulated RAW264.7 macrophages [38]. However, the mechanism underlying the anti‐inflammatory effects of derrisfolin A remains unclear. During our study to characterize derrisfolin A, we found that derrisfolin A upregulates the expression of RGN. In this study, we performed experiments to determine whether derrisfolin A‐mediated and gene transfer‐mediated RGN induction reduced LPS‐induced inflammatory responses in pancreatic MIN6 β‐cells. We also co‐cultured MIN6 cells with RAW264.7 macrophages to elucidate whether derrisfolin A‐mediated and gene transfer‐mediated RGN induction in RAW264.7 macrophages blocks the incidence of LPS‐induced paracrine effect of RAW264.7 macrophages on MIN6 cell inflammation.

## Materials and methods

### Materials

The following materials from the indicated sources were used in this study: Dulbecco’s modified Eagle’s medium (DMEM), Roswell Park Memorial Institute (RPMI) 1640 medium, FBS, penicillin, streptomycin, TRIzol Reagent, SYBR GreenER qPCR SuperMix, 5‐(and‐6)‐chloromethyl‐2′,7′‐dichlorodihydrofluorescein diacetate acetyl ester (CM‐H_2_DCFDA), Micro BCA protein assay kit, and SuperBlock blocking buffer from Thermo Fisher Scientific (Waltham, MA, USA)**;** RNase‐free DNase from Qiagen (Chatsworth, CA, USA); LPS, 2‐(4‐carboxyphenyl)‐4,4,5,5‐tetramethylimidazoline‐1‐oxyl‐3‐oxide (c‐PTIO), Hoechst 33342 dye, and rabbit polyclonal RGN antibody from Millipore Sigma (St. Louis, MO, USA); lentiviral vectors containing a single‐guide RNA (sgRNA)/CRISPR/Cas9 All‐in‐One gene targeting system from Applied Biological Materials (Richmond, BC, Canada); ViroMag R/L viral gene delivery reagent from OZ Biosciences (Marseille, France); IL‐1β neutralizing antibody, TNF‐α neutralizing antibody, IL‐1β ELISA kits, and TNF‐α ELISA kits from R & D Systems (Minneapolis, MN, USA); NO assay kits from Dojin (Kumamoto, Japan); NF‐κB reporter vector pGL4.32[*luc2P*/NF‐κB‐RE/Hygro], pRL‐TK vector, and a Dual‐Glo luciferase assay kit from Promega (Madison, WI, USA); mouse monoclonal β‐actin antibody from Cell Signaling Technology (Danvers, MA, USA); peroxidase‐conjugated donkey anti‐rabbit IgG and anti‐mouse IgG antibody, and enhanced chemiluminescence (ECL) western blotting detection reagents from GE Healthcare Life Sciences (Amersham, UK); Can Get Signal Solution from Toyobo (Osaka, Japan); OxiSelect Nitrotyrosine ELISA kits from Cell Biolabs, Inc. (San Diego, CA, USA); lipid peroxidation assay kits from BioVision, Inc. (Milpitas, CA, USA); protease and phosphatase inhibitor cocktail tablets, and terminal deoxynucleotidyl transferase biotin‐dUTP nick end labeling (TUNEL) *in situ* apoptosis detection kits from Roche (Mannheim, Germany); Millicell Hanging Cell Culture Inserts (pore size, 0.4 µm; polyethylene terephthalate membrane) from Millipore (Billerica, MA, USA); and VECTASHIELD HardSet Antifade mounting medium from Vector Laboratories (Burlingame, CA, USA).

### Cell culture and treatment

MIN6 mouse pancreatic β cell line [39] was kindly provided by J.‐I. Miyazaki (Osaka University, Japan). MIN6 cells were maintained in DMEM supplemented with 15% heat‐inactivated FBS, 70 μm β‐mercaptoethanol, 50 U·mL^−1^ penicillin, and 50 μg·mL^−1^ streptomycin in a humidified atmosphere of 5% CO_2_ at 37 °C. The RAW264.7 mouse macrophage cell line was maintained in RPMI 1640 medium containing 10% heat‐inactivated FBS, 50 U·mL^−1^ penicillin, and 50 μg·mL^−1^ streptomycin under a humidified atmosphere of 5% CO_2_ at 37 °C. The derrisfolin A used in this study was isolated and purified as previously described [38]. Derrisfolin A purity was assessed by thin‐layer chromatography and ^13^C nuclear magnetic resonance. Derrisfolin A was dissolved in DMSO and added to a complete medium to yield a final DMSO concentration of 0.5% (v/v), which did not affect cell viability.

### Immunoblot analysis of RGN expression

Cells were lysed in RIPA lysis buffer containing the protease inhibitor mixture. The lysed cells were then centrifuged at 15 000 ***g*** for 15 min at 4 °C, and the supernatants were collected for western blotting. The supernatant proteins were resolved using 12% sodium dodecyl sulfate‐polyacrylamide gel electrophoresis and were transferred to polyvinylidene difluoride membranes. After blocking for 1 h in SuperBlock blocking buffer, the membranes were incubated with rabbit anti‐RGN antibody (1 : 1000) in Can Get Signal Solution 1 and then with peroxidase‐conjugated donkey anti‐rabbit IgG antibody in Can Get Signal Solution 2. The bound antibody was visualized via ECL. β‐actin was used as the loading control, and the blots were probed with anti‐β‐actin antibody to normalize the protein levels. Primary antibodies were used at 1 : 500 dilution, and secondary antibodies were used at 1 : 5000 dilution.

### Virus infection for RGN‐KO and overexpression

We prepared RGN‐KO MIN6 cells and RAW264.7 macrophages using CRISPR/Cas9/sgRNA technology. The sgRNA sequences targeting RGN were 5ʹ‐TTACGGGAGAACTACAGGTG‐3ʹ, 5ʹ‐TGTGACGCTTCCTCCCATAC‐3ʹ, and 5ʹ‐TCAAGTGCAGCGAGTTGCTG‐3ʹ. A scramble sgRNA sequence was used as the control sgRNA. A mixture of three types of lentiviruses expressing sgRNA against RGN was used to transduce MIN6 cells and RAW264.7 macrophages. We prepared stable RGN‐overexpressing MIN6 cells and RAW264.7 macrophages by generating a retrovirus harboring RGN or β‐galactosidase (LacZ) expression using a previously described method [2]. The lentivirus or retrovirus was infected into MIN6 cells and RAW264.7 macrophages in the presence of ViroMag R/L viral gene delivery reagent. Cells infected with lentivirus or retrovirus were selected with puromycin for use in subsequent experiments.

### Quantitative RT‐PCR analysis

Total RNA was prepared from cultured cells using TRIzol reagent according to the manufacturer’s instructions and then treated with RNase‐free DNase. cDNA was synthesized from the total RNA using a high capacity cDNA synthesis kit. Quantitative PCR was performed in a reaction mixture comprising cDNA, specific sense and antisense primers, and SYBR GreenER qPCR SuperMix on a LightCycler 480 System II (Roche, Basel, Switzerland). The relative quantity of target mRNA was calculated using the comparative cycle threshold method and was normalized using β‐actin as an endogenous control. The nucleotide sequences of the specific primers used were as follows: 5′‐AAGAGATGTTGAACTATGTCC‐3′ (sense) and 5′‐CCTGGCTAGTGCTTCAGACT‐3′ (antisense) for *Nos2*; 5′‐AGAAGAGCCCATCCTCTGTGACTC‐3′ (sense) and 5′‐GTACAAAGCTCATGGAGAATATCA‐3′ (antisense) for *Il1b*; 5′‐GCCTCCCTCTCATCAGTTCTATGG‐3′ (sense) and 5′‐TCCAGCTGCTCCTCCACTTGGTGG‐3′ (antisense) for *Tnf*; and 5′‐GTGGGCCGCCCTAGGCACCA‐3′ (sense) and 5′‐GGTTGGCCTTAGGGTTCAGG‐3′ (antisense) for *Actb*.

### Assay of NF‐κB transcriptional activity

The transcriptional activity of NF‐*κ*B was determined by a reporter gene assay, as previously described [2]. Briefly, cells were co‐transfected with an NF‐κB‐dependent firefly luciferase reporter vector or a negative control vector along with internal control vector pRL‐TK (thymidine kinase promoter‐*Renilla* luciferase reporter vector) using lipofectamine reagent. After transfection, the cells were lysed in reporter buffer, and the luciferase activity of the lysates was measured using Dual‐Glo luciferase assay kit, according to the manufacturer’s instructions.

### Co‐culture of MIN6 cells with RAW264.7 macrophages

MIN6 cells were co‐cultured with RAW264.7 macrophages in a transwell culture system. Briefly, the MIN6 cells were seeded on the bottom of a 24‐well plate, and RAW264.7 macrophages were seeded on 24‐well Millicell Hanging Culture Inserts set in another 24‐well plate. Following overnight culture in their respective maintenance medium, the MIN6 cells were co‐cultured with the RAW264.7 macrophages by transferring the culture inserts into the 24‐well plates containing the MIN6 cells. The RAW264.7 macrophage culture inserts were removed after the co‐culture experiment, and the treated MIN6 cells were used for subsequent assays. The culture of MIN6 cells alone was performed on the bottom of 24‐well plates, and the culture of RAW264.7 macrophages alone was performed on culture inserts set in a 24‐well plate. If required, NO scavenger c‐PTIO, a mixture of neutralizing antibodies against IL‐1β and TNF‐α, or isotype control IgG was added to the culture medium.

### Assay of apoptotic cells

Apoptosis of MIN6 cells was estimated using a TUNEL *in situ* apoptosis detection kit. The MIN6 cells were treated on a round cover glass at the bottom of a 24‐well plate. The cells were fixed with 4% paraformaldehyde for 10 min, permeabilized using 0.1% Triton X‐100 for 10 min, and then incubated with the TUNEL reaction mixture for 1 h at 37 °C. The total cell counts were determined following nuclear counterstaining with Hoechst 33342 dye (10 μg·mL^−1^). The number of TUNEL‐positive cells was counted in randomly selected fields using a Zeiss LSM800 confocal microscope (Carl Zeiss MicroImaging GmbH, Jena, Germany).

### Measurement of proinflammatory mediator production

After cell treatment, the cell culture medium was collected to measure the secretion levels of NO, IL‐1β, and TNF‐α. The NO concentration was measured using Griess reagent from the NO^2–^/NO^3–^ assay kit. The IL‐1β and TNF‐α concentrations were measured by ELISA.

### Measurement of intracellular reactive oxygen species

The fluorescent dye CM‐H_2_DCFDA was used to visualize intracellular reactive oxygen species (ROS) generation in MIN6 cells. The MIN6 cells were treated on a round cover glass at the bottom of a 24‐well plate. Thereafter, the cells were incubated in culture medium containing CM‐H_2_DCFDA (5 μm) for 30 min, fixed with 4% formaldehyde in Dulbecco’s phosphate‐buffered saline, and mounted with VECTASHIELD HardSet Antifade Mounting Medium with DAPI. The cells were visualized using a Zeiss LSM800 confocal microscope, and images were taken of randomly selected fields. The fluorescence intensities of CM‐H_2_DCFDA were quantified using carl zeiss microimaging software.

### Lipid peroxidation assay

The malondialdehyde (MAD) content of the MIN6 cells was measured as an indicator of oxidative stress using a lipid peroxidation assay kit, as previously described [2]. Briefly, the cells were sonicated for 20 s in MAD lysis buffer. After centrifugation at 13 000 ***g*** for 10 min, the supernatants were incubated with thiobarbituric acid at 95 °C for 1 h. Subsequently, sample fluorescence was measured using a PerkinElmer microplate spectrofluorometer (EnSpire, Norwalk, CT, USA). The MAD contents were normalized to total protein, which was determined using a Micro BCA protein assay kit.

### Measurement of 3‐nitrotyrosine content

The 3‐nitrotyrosine (3‐NT) content in MIN6 cells was measured as an indicator of nitrosative stress using an OxiSelect Nitrotyrosine ELISA kit, as previously described [2]. Briefly, the cells were lysed in RIPA buffer containing protease and phosphatase inhibitor mixtures for 15 min on ice. After centrifugation at 15 000 ***g*** for 15 min at 4 °C, the supernatants were collected, and the 3‐NT contents were normalized to total protein, which was determined using a Micro BCA protein assay kit.

### Statistical analysis

All data were presented as means ± SE of three independent experiments in which each measurement was performed in triplicate. The differences among multiple groups were estimated by one‐way ANOVA followed by Tukey’s *post hoc* test. A *P*‐value < 0.05 was considered significant. All analyses were performed using graphpad prism 7.0 software (GraphPad Software, San Diego, CA, USA).

## Results

### Derrisfolin A inhibited LPS‐induced proinflammatory response gene expression in pancreatic MIN6 β‐cells and RAW264.7 macrophages by inducing RGN expression

**Fig. 1:**
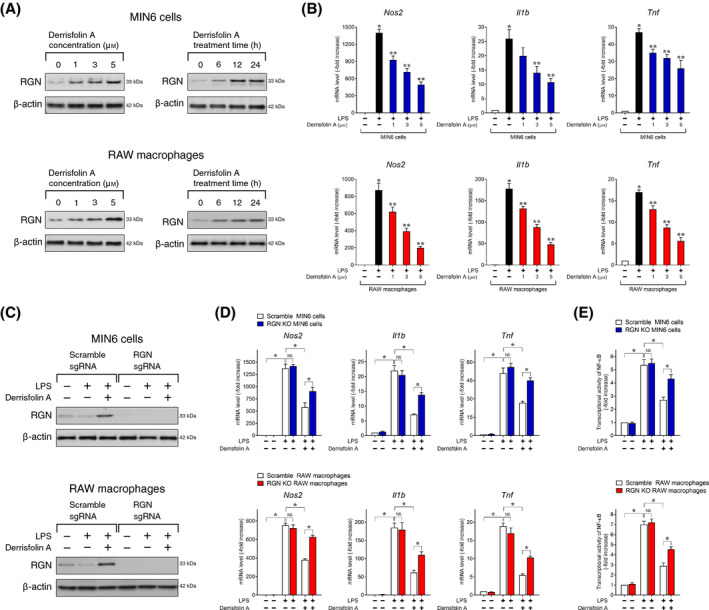
Derrisfolin A, a chemical inducer of RGN, inhibits LPS‐induced expression of proinflammatory response genes in pancreatic MIN6 β‐cells and RAW264.7 macrophages. (A) MIN6 cells and RAW264.7 macrophages were treated with the indicated concentrations of derrisfolin A for the indicated periods. Thereafter, the cells were lysed and then processed for western blotting analysis using anti‐RGN antibody. β‐actin was used as the loading control. (B) MIN6 cells or RAW264.7 macrophages were pretreated with or without the indicated concentrations of derrisfolin A for 12 h, followed by treatment with or without LPS (100 ng·mL^−1^) for an additional 18 h. Total RNA was extracted and processed for real‐time RT‐PCR analysis using primer pairs for *Nos2, Il1b*, and *Tnf*. Data are presented as fold increases compared with the mRNA level in untreated cells, which was set at 1.0. Data are presented as means ± SE from three independent experiments performed in triplicate. One‐way ANOVA followed by Tukey’s *post hoc* test was performed to compare significance differences among groups. *P and **P < 0.05 were considered significant. *P < 0.05, LPS‐treated cells vs. untreated cells. **P < 0.05, derrisfolin A‐pretreated, LPS‐treated cells vs. LPS‐treated cells. (C) Using a CRISPR/Cas9 system with lentiviruses harboring control scrambled sgRNA or sgRNA targeting RGN, scramble control or RGN knockout (KO) MIN6 cells and RAW264.7 macrophages were generated. The cells were pretreated with or without derrisfolin A (5 μm) for 12 h, followed by treatment with or without LPS (100 ng·mL^−1^) for an additional 24 h and then processed for western blotting analysis using anti‐RGN antibody. β‐actin was used as the loading control. (D) The scramble control or RGN‐KO MIN6 cells or RAW264.7 macrophages were pretreated with or without derrisfolin A (5 μm) for 12 h followed by treatment with or without LPS (100 ng·mL^−1^) for an additional 18 h and then processed for real‐time RT‐PCR analysis using primer pairs for *Nos2*, *Il1b*, and *Tnf*. Data are presented as fold increases compared with untreated scramble control cells, which was set at 1.0. Data are presented as means ± SE from three independent experiments performed in triplicate. One‐way ANOVA followed by Tukey’s *post hoc* test was performed to compare significance differences among groups. *P < 0.05 was considered statistically significant. NS, not significant. (E) The scramble control or RGN‐KO cells were transiently co‐transfected with an NF‐κB‐responsive reporter vector and an internal control vector. After 8 h, the transfected cells were pretreated with or without derrisfolin A (5 μm) for 12 h, followed by treatment with or without LPS (100 ng·mL^−1^) for an additional 18 h. Then, they were lysed for NF‐κB reporter assays using a dual‐luciferase reporter assay system. Data are presented as fold increases compared with untreated scramble control cells, which was set at 1.0. Data are presented as means ± SE from three independent experiments performed in triplicate. One‐way ANOVA followed by Tukey’s *post hoc* test was performed to compare significance differences among groups. *P < 0.05 was considered statistically significant. NS, not significant.

We previously reported that derrisfolin A (Fig. S1), our isolated novel natural compound, inhibited the excess production of the proinflammatory mediator NO in RAW264.7 macrophages stimulated with LPS and interferon‐γ [38]. We used microarray analysis to determine changes in gene expression in derrisfolin A‐treated MIN6 cells and RAW264.7 macrophages, and we found that RGN gene expression was dramatically upregulated among 12 differentially expressed genes, with > 10‐fold changes in the two‐type cells (unpublished data). Derrisfolin A was capable of increasing the expression of endogenous RGN protein in MIN6 cells and RAW264.7 macrophages in a concentration‐ and time‐dependent manner (Fig. 1A). Furthermore, derrisfolin A concentration‐dependently inhibited LPS‐induced mRNA expression of proinflammatory response genes *Nos2*, *Il1b*, and *Tnf* in MIN6 cells and RAW264.7 macrophages (Fig. 1B). To determine whether derrisfolin A exhibited an anti‐inflammatory effect by inducing RGN expression, we used the CRISPR/Cas9 technique to disrupt the RGN gene in MIN6 cells and RAW264.7 macrophages and then examined the KO effect of the RGN gene on derrisfolin A‐meditated RGN induction (Fig. 1C). The immunoblot analysis of MIN6 cells and RAW264.7 macrophages expressing control scrambled sgRNA showed a few endogenous RGN proteins (lane 1) and a slight but insignificant decrease in RGN expression following LPS treatment (lane 2). Furthermore, derrisfolin A induced RGN expression upon LPS treatment (lane 3). In contrast, endogenous RGN protein was not detected in MIN6 cells or RAW264.7 macrophages expressing sgRNA targeting RGN (lane 4), indicating successful RGN‐KO in two‐type cells. Additionally, we confirmed that endogenous RGN was not expressed upon treatment with LPS alone (lane 5) and derrisfolin A plus LPS (lane 6) in the two‐type cells, indicating the block of derrisfolin A‐mediated RGN induction in MIN6 cells and RAW264.3 macrophages expressing sgRNA targeting RGN.

Next, we asked whether RGN‐KO affected the ability of derrisfolin A to inhibit LPS‐induced mRNA expression of *Nos2*, *Il1b*, and *Tnf* in MIN6 cells and RAW264.7 macrophages (Fig. 1D). In the scramble control MIN6 cells, the mRNA levels of *Nos2*, *Il1b*, and *Tnf* were significantly increased by LPS treatment, and derrisfolin pretreatment attenuated LPS‐induced increases in mRNA levels of the three proinflammatory genes. The LPS‐induced mRNA level of the three proinflammatory genes in RGN‐KO MIN6 cells did not differ significantly from that in the scramble control MIN6 cells, indicating that RGN loss in MIN6 cells did not affect LPS‐induced expression of proinflammatory genes. Interestingly, after pretreatment with derrisfolin A, RGN‐KO MIN6 cells had higher levels of LPS‐induced mRNA expression of the three proinflammatory genes relative to the scramble control MIN6 cells, indicating that RGN deficiency in MIN6 cells attenuated the inhibitory effect of derrisfolin A on mRNA expression of the three proinflammatory genes. Similar to RGN‐KO MIN6 cells, RGN‐KO RAW264.7 macrophages also showed a significant decrease in the inhibitory effect of derrisfolin A on LPS‐induced mRNA expression of *Nos2*, *Il1b*, and *Tnf* (Fig. 1D). Because LPS‐mediated mRNA upregulation of *Nos2, Il1b*, and *Tnf* is necessary to activate transcription factor NF‐κB [24, 25, 26], we further examined whether derrisfolin A, which is a chemical inducer of RGN, inhibits LPS‐mediated NF‐κB activation in MIN6 cells and RAW264.7 macrophages (Fig. 1E). In the two‐type cells, there was no significant difference in the LPS‐induced increase in NF‐κB transcriptional activity between the scramble control and RGN‐KO cells, while the inhibitory effect of derrisfolin A on LPS‐induced NF‐κB transcriptional activity was significantly lower in the RGN‐ KO cells than in the scramble control cells. Taken together, these results suggested that derrisfolin A‐mediated RGN induction in both MIN6 cells and RAW264.7 macrophages could exert an inhibitory effect on LPS‐induced proinflammatory response gene expression by ameliorating NF‐κB activation.

**Fig. 2:**
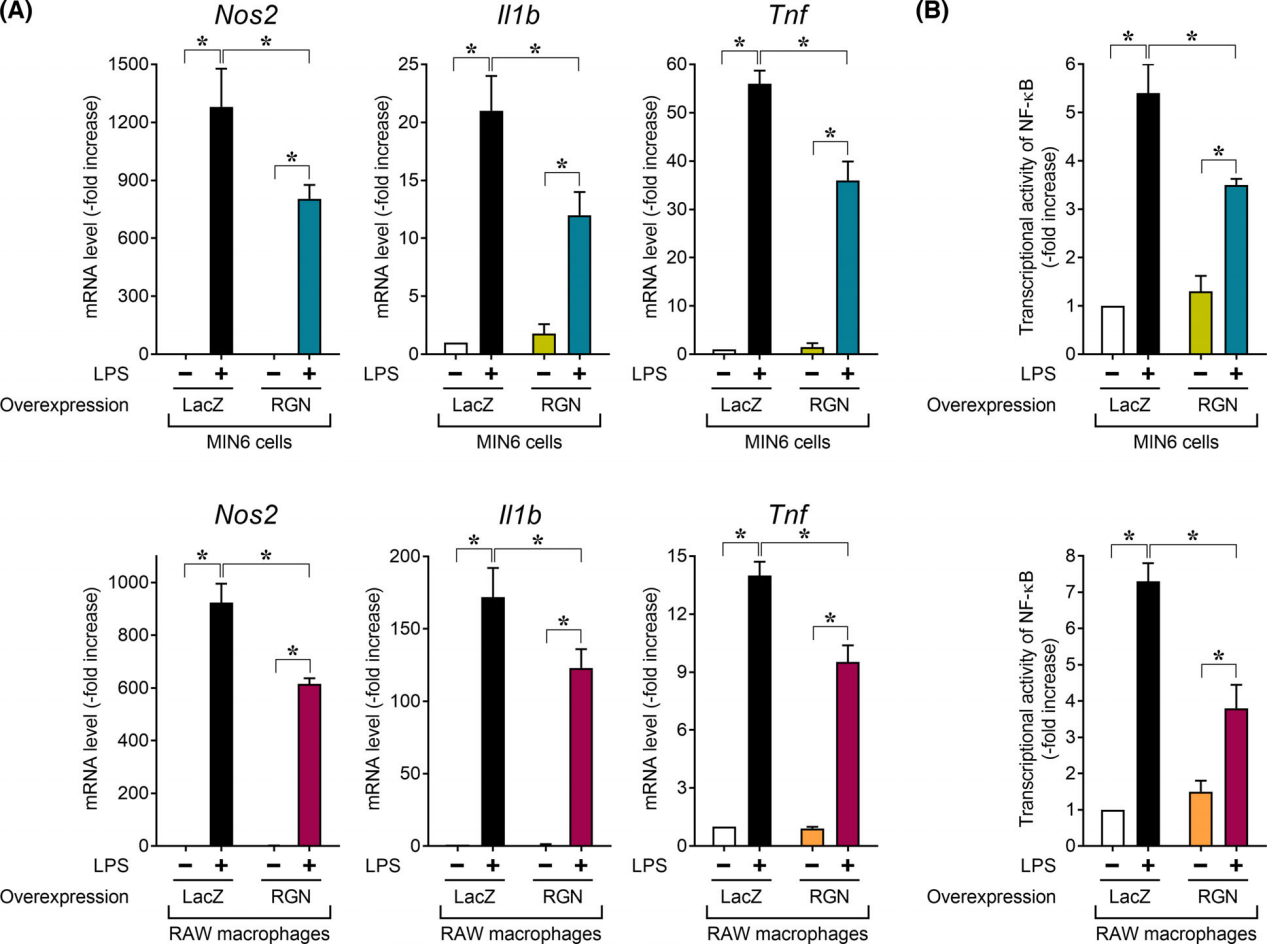
RGN overexpression inhibited LPS‐induced expression of proinflammatory response genes in pancreatic MIN6 β‐cells and RAW264.7 macrophages. The retrovirus‐mediated gene transfer method was used to generate β‐galactosidase (LacZ)‐overexpressing or RGN‐overexpressing MIN6 cells and RAW264.7 macrophages. The cells overexpressing LacZ or RGN were treated with or without LPS (100 ng·mL^−1^) for 18 h and then processed for real‐time RT‐PCR analysis using primer pairs for *Nos2*, *Il1b*, and *Tnf* (A) and NF‐κB reporter assays (B). The levels of proinflammatory gene expression and NF‐κB transcriptional activity were measured as described in the Fig. 1 legend. Data are presented as means ± SE from three independent experiments performed in triplicate. One‐way ANOVA followed by Tukey’s *post hoc* test was performed to compare significance differences among groups.*P < 0.05 was considered statistically significant.

To further identify the effectivity of RGN induction itself against LPS‐induced inflammation in MIN6 cells and RAW264.7 macrophages, we generated LacZ‐ or RGN‐overexpressing MIN6 cells and RAW264.7 macrophages using a retrovirus‐mediated gene transfer system and then examined the effect of RGN overexpression on LPS‐induced proinflammatory response gene expression in the two‐type cells (Fig. 2A). In MIN6 cells and RAW264.7 macrophages, following LPS treatment, RGN overexpression resulted in a significant decrease in LPS‐induced mRNA expression of *Nos2*, *Il1b*, and *Tnf* relative to LacZ overexpression. Additionally, in the two‐type cells, RGN overexpression showed significantly decreased LPS‐induced NF‐κB transcription activity compared with LacZ overexpression (Fig. 2B). These results indicated that RGN overexpression in MIN6 cells and RAW264.7 macrophages was effective in suppressing LPS‐induced proinflammatory response gene expression via NF‐κB activation. The RGN overexpression data described above supported the effectivity of derrisfolin A‐mediated RGN induction itself against LPS‐induced inflammatory responses in both MIN6 cells and RAW264.7 macrophages.

### Derrisfolin A attenuated apoptosis and oxidative/nitrosative stress in pancreatic MIN6 β‐cells treated with LPS

The long‐term treatment of MIN6 cells with LPS alone resulted in a significant increase in the number of apoptotic cells (Fig. S2A). Additionally, an LPS‐induced increase in the amounts of NO, IL‐1β, and TNF‐α in the culture medium was observed in the MIN6 alone culture (Fig. S2B). Furthermore, we confirmed a significant decrease in LPS‐induced apoptotic cell number with the addition of NO scavenger c‐PTIO or a mixture of neutralizing antibodies in the MIN6 alone culture (Fig. S2C), indicating that NO, IL‐1β, and TNF‐α cause apoptosis in LPS‐treated MIN6 cells in an autocrine fashion.

**Fig. 3:**
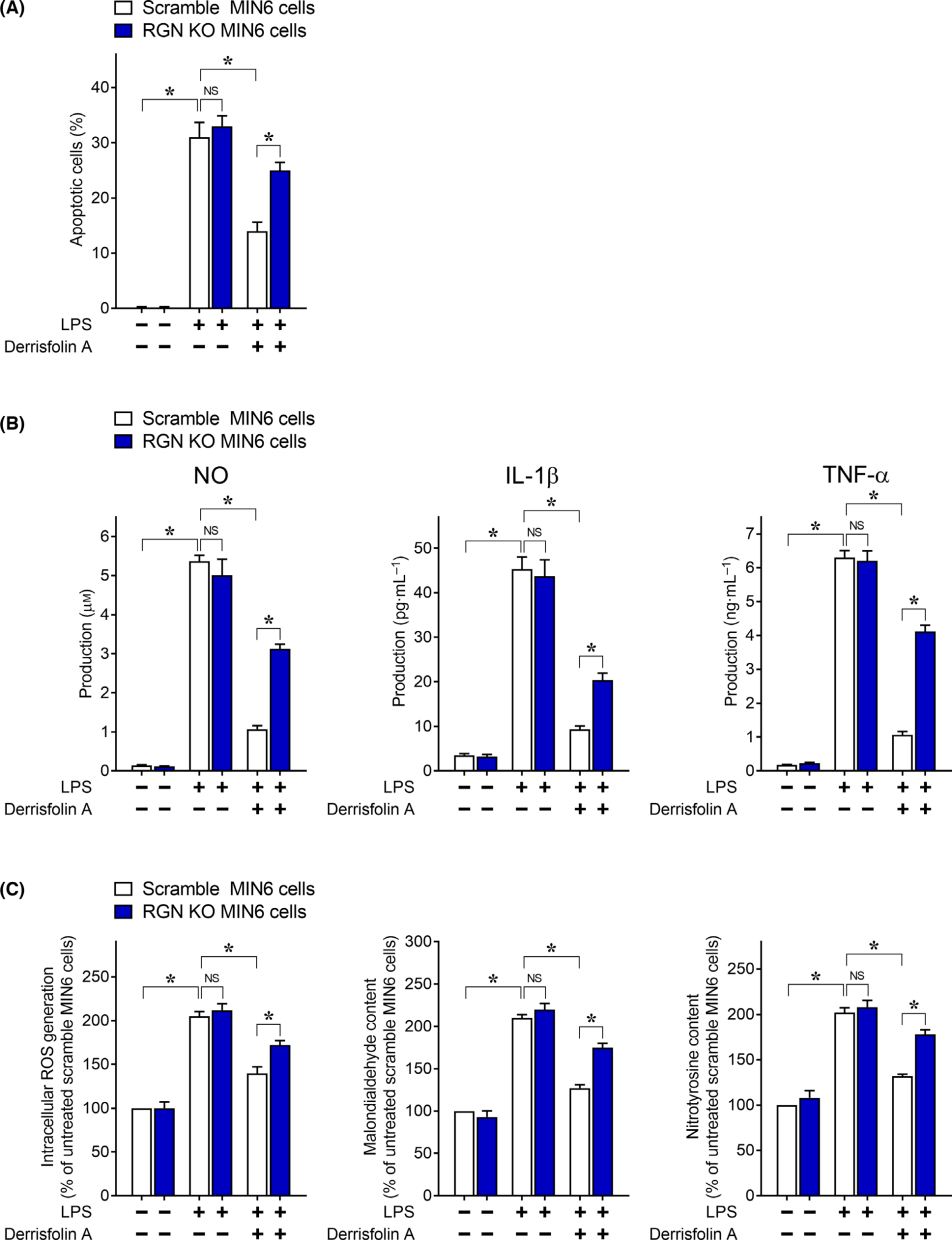
Derrisfolin A inhibited LPS‐induced inflammatory cytotoxicity in MIN6 cells. The scramble control or RGN‐KO MIN6 cells were pretreated with or without derrisfolin A for 12 h followed by treatment with or without LPS (100 ng·mL^−1^) for an additional 36 h. (A) The cells were incubated with the TUNEL reaction mixture to determine the rates of apoptosis, and TUNEL‐positive apoptotic nuclei were identified under confocal microscopy. (B) The concentration of nitric oxide (NO) in the cell culture medium was measured using the Griess reaction method, and the concentrations of IL‐1β and TNF‐α in the cell culture medium were measured using ELISA. (C) Intracellular ROS generation was detected using the fluorescent indicator CM‐H_2_DCFDA. Oxidative damage was estimated by measuring the malondialdehyde content as an index of lipid peroxidation. Nitrosative damage was estimated by measuring the content of 3‐NT, which indicates the nitration of tyrosine by peroxynitrite (ONOO^−^) generated from NO and $O_{2}^{‐}$. All data are presented as means ± SE from three independent experiments performed in triplicate. One‐way ANOVA followed by Tukey’s *post hoc* test was performed to compare significance differences among groups. *P < 0.05 was considered statistically significant. NS, not significant.

We initially examined the effect of derrisfolin A, a chemical inducer of RGN, on LPS‐induced MIN6 cell apoptosis (Fig. 3A). LPS showed a significant increase in apoptotic cells in the scramble control MIN6 cells, and pretreatment with derrisfolin A reduced the number of LPS‐induced apoptotic cells, indicating the inhibitory effect of derrisfolin A on LPS‐induced MIN6 cell apoptosis. Additionally, there was no significant difference in the number of LPS‐induced apoptotic cells between scramble control and RGN‐KO MIN6 cells, indicating that RGN deficiency did not alter LPS‐induced MIN6 cell apoptosis. Interestingly, after pretreatment with derrisfolin A, the number of apoptotic RGN‐KO MIN6 cells was significantly higher than the number of apoptotic scramble control MIN6 cells, indicating that RGN deficiency attenuated the inhibitory effect of derrisfolin A on LPS‐induced MIN6 cell apoptosis. In addition, derrisfolin A attenuated the induction of mitochondrial dysfunction‐mediated apoptosis by LPS, which was characterized by a loss of mitochondrial membrane potential and activation of downstream caspase‐9/caspase‐3 (data not shown). These results suggested that derrisfolin A inhibited LPS‐induced MIN6 cell apoptosis by inducing RGN expression.

Next, we examined the effect of derrisfolin A on the levels of proinflammatory mediators secreted from LPS‐treated MIN6 cells into the cell culture medium (Fig. 3B). Treatment of scramble control MIN6 cells with LPS showed increased levels of NO, IL‐1β, and TNF‐α in the medium, and the LPS‐induced production of the three proinflammatory mediators was inhibited by pretreatment with derrisfolin A. Additionally, LPS‐induced production of the three proinflammatory mediators in RGN‐KO MIN6 cells did not differ from that in scramble control cells, but the inhibitory effect of derrisfolin A on LPS‐induced production of the three proinflammatory mediators was lower in RGN‐KO MIN6 cells compared with scramble control cells. Considering the protective effect of NO scavenger c‐PTIO or a mixture of neutralizing antibodies against IL‐1β and TNF‐α on LPS‐induced MIN6 cell apoptosis (Fig. S2C), these results suggested that derrisfolin A inhibited the production of the three proinflammatory mediators in MIN6 cells by inducing RGN expression, resulting in decreased apoptosis of MIN6 cells by the three autocrine mediators.

Because cytokines such as IL‐1β and TNF‐α trigger oxidative/nitrosative stress in pancreatic β‐cells, leading to β‐cell dysfunction [40], we examined the effect of derrisfolin A on oxidative/nitrosative stress damage in LPS‐treated MIN6 cells (Fig. 3C). We determined the amount of intracellular ROS generation and the amount of MAD, a metabolite of ROS‐mediated lipid peroxidation, to estimate oxidative stress damage. Because *Nos2*‐derived excessive NO reacts with $O_{2}^{‐}$ to produce the powerful oxidant ONOO^−^, which can lead to the nitration of tyrosine residues in proteins, 3‐NT was used as a biomarker of nitrosative stress. We observed a significant increase in the levels of ROS generation, MAD content, and 3‐NT content in scramble control MIN6 cells following LPS treatment, and this increase was inhibited by derrisfolin A pretreatment, indicating the inhibitory effect of derrisfolin A on LPS‐induced oxidative/nitrosative stress in MIN6 cells. Additionally, no significant differences in LPS‐induced levels of ROS generation, MAD content, and 3‐NT content were observed between scramble control and RGN‐KO MIN6 cells. However, the levels of ROS generation, MAD content, and 3‐NT content in RGN‐KO MIN6 cells were higher than those in the scramble control MIN6 cells following derrisfolin A pretreatment, indicating that RGN loss reduced the inhibitory effect of derrisfolin A on LPS‐induced oxidative/nitrosative stress in MIN6 cells. These findings suggested that derrisfolin A attenuated LPS‐induced oxidative/nitrosative stress damage in MIN6 cells by inducing RGN expression.

**Fig. 4:**
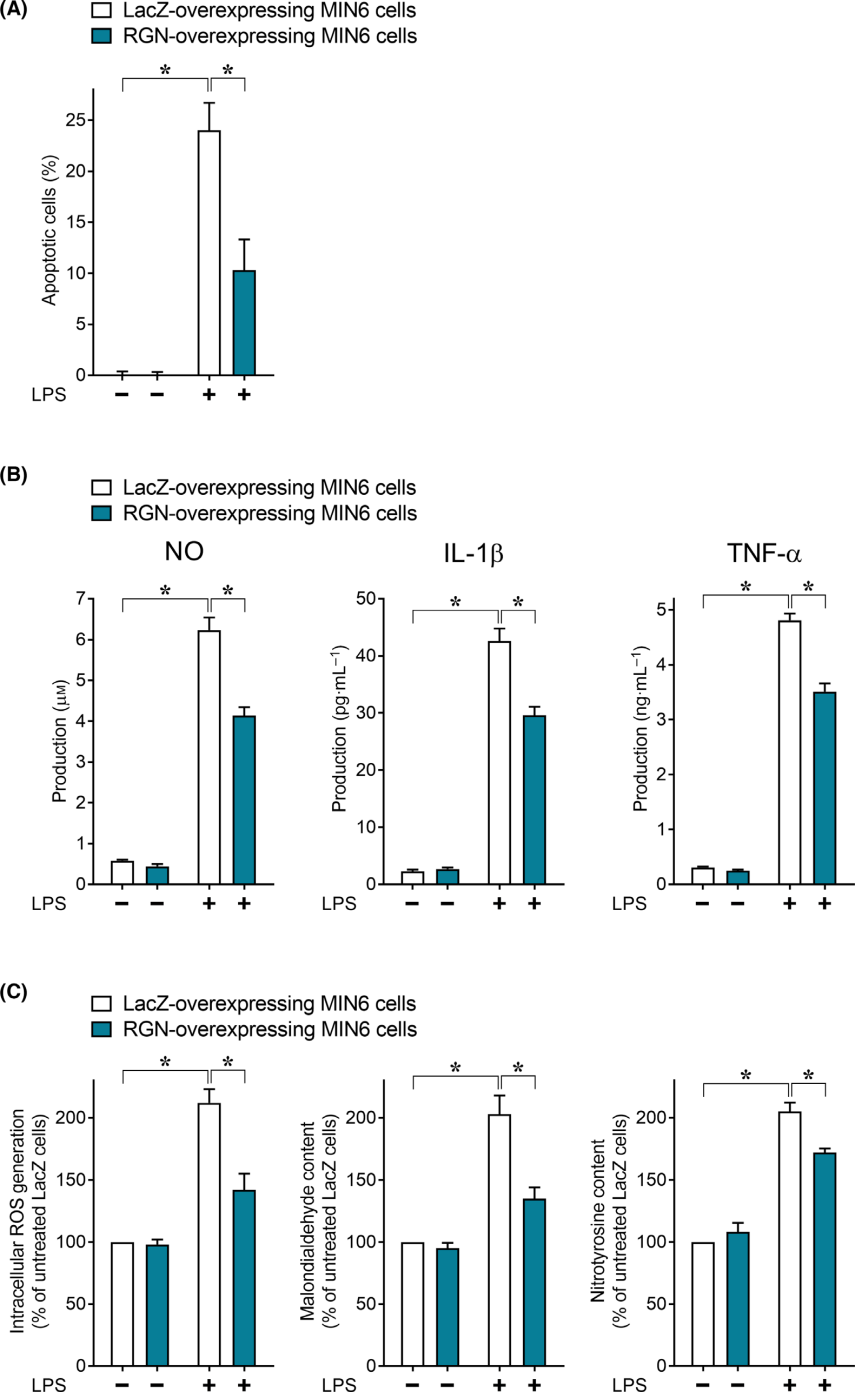
RGN overexpression inhibited LPS‐induced inflammatory cytotoxicity in MIN6 cells. MIN6 cells overexpressing LacZ or RGN were treated with or without LPS (100 ng·mL^−1^) for 36 h and then processed for assay of the number of apoptotic cells (A); the level of proinflammatory mediators in the culture medium (B); and intracellular ROS generation, malondialdehyde content, and 3‐NT content (C). Each assay was performed as described in the Fig. 3 legend. All data are presented as means ± SE from three independent experiments performed in triplicate. One‐way ANOVA followed by Tukey’s *post hoc* test was performed to compare significance differences among groups. *P < 0.05 was considered statistically significant.

We examined the effect of RGN overexpression on LPS‐induced apoptosis and oxidative/nitrosative stress in MIN6 cells to elucidate the effectiveness of RGN induction itself toward LPS‐induced inflammatory cytotoxicity in MIN6 cells. As shown in Fig. 4A,B, RGN‐overexpressing MIN6 cells resulted in a significant decrease in LPS‐induced apoptosis after LPS treatment relative to LacZ‐overexpressing MIN6 cells, accompanied by decreased production of NO, TNF‐α, and IL‐1β, suggesting that RGN overexpression protects MIN6 cells from apoptosis induced by the three autocrine proinflammatory mediators. Additionally, following LPS treatment, RGN‐overexpressing MIN6 cells exhibited significant decreases in the level of intracellular ROS generation, MAD content, and 3‐NT content compared with LacZ‐overexpressing control cells (Fig. 4C), suggesting that RGN overexpression attenuates LPS‐induced oxidative/nitrosative stress damage in MIN6 cells. These RGN overexpression experiments supported the effectivity of RGN induction itself by derrisfolin A against LPS‐induced inflammatory cytotoxicity in MIN6 cells.

### Derrisfolin A attenuated the incidence of LPS‐induced paracrine effect of RAW264.7 macrophages on apoptosis and oxidative/nitrosative stress in pancreatic MIN6 β‐cells in the co‐culture conditions

Upon LPS treatment, co‐culture with RAW264.7 macrophages significantly enhanced the number of apoptotic MIN6 cells in comparison with the results obtained in the MIN6 cell alone culture (Fig. S2A). Additionally, co‐culture with RAW264.7 macrophages increased the levels of NO, TNF‐α, and IL‐1β in the culture medium after LPS treatment in comparison with the results obtained in the MIN6 cell alone culture (Fig. S2B), indicating that the three proinflammatory mediators released from LPS‐stimulated RAW264.7 macrophages also contributed to increasing the levels of the three proinflammatory mediators in the co‐culture medium. Furthermore, we confirmed that the addition of NO scavenger c‐PTIO or a mixture of neutralizing antibodies against IL‐1β and TNF‐α decreased the number of apoptotic MIN6 cells enhanced by co‐culture with RAW264.7 macrophages in the presence of LPS (Fig. S2C), indicating that the LPS‐induced paracrine effect of RAW264.7 macrophages on MIN6 cell apoptosis existed in the co‐culture conditions.

**Fig. 5:**
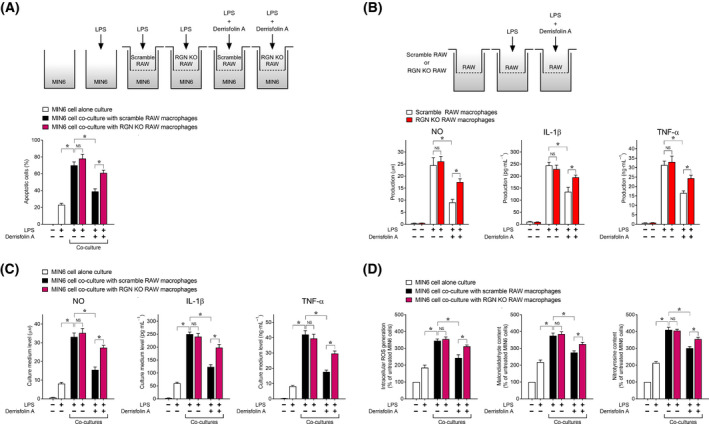
Derrisfolin A inhibited the incidence of LPS‐induced paracrine effects of RAW264.7 macrophages on inflammatory cytotoxicity in MIN6 cells under co‐culture conditions. (A) MIN6 cells were seeded in the bottom of a 24‐well plate with or without scramble control or RGN‐KO RAW264.7 macrophages in the inserts. The cells were pretreated with or without derrisfolin A (5 μm) for 12 h followed by treatment with or without LPS (100 ng·mL^−1^) for an additional 36 h and then processed to assay the number of apoptotic cells. (B) The scramble control or RGN‐KO RAW264.7 macrophages were pretreated with or without derrisfolin A (5 μm) for 12 h followed by treatment with or without LPS (100 ng·mL^−1^) for an additional 36 h and then processed to assay the amounts of NO, IL‐1β, and TNF‐α in the culture medium. (C, D) MIN6 cells were seeded in the bottom of a 24‐well plate with or without scramble control or RGN‐KO RAW264.7 macrophages in the inserts. The cells were pretreated with or without derrisfolin A (5 μm) for 12 h, followed by treatment with or without LPS (100 ng·mL^−1^) for an additional 36 h and then processed to assay the levels of proinflammatory mediators in the culture medium (C) as well as intracellular ROS generation, malondialdehyde content, and 3‐NT content (D). Each assay was performed as described in the Fig. 3 legend. All data are presented as means ± SE from three independent experiments performed in triplicate. One‐way ANOVA followed by Tukey’s *post hoc* test was performed to compare significance differences among groups. *P < 0.05 was considered statistically significant. NS, not significant.

We examined whether derrisfolin A inhibits the incidence of LPS‐induced paracrine effect of RAW264.7 macrophages on MIN6 cell apoptosis in the co‐culture condition (Fig. 5A). Upon LPS treatment, co‐culture with scramble control RAW264.7 macrophages enhanced the number of apoptotic MIN6 cells even more in comparison with the MIN6 cell alone culture. Additionally, following LPS treatment, co‐culture with RGN‐KO RAW264.7 macrophages did not significantly increase the number of apoptotic MIN6 cells relative to co‐culture with scramble control RAW264.7 macrophages, indicating that RGN deficiency in RAW264.7 macrophages did not affect co‐culture‐induced MIN6 cell apoptosis. Interestingly, following derrisfolin A pretreatment, the number of apoptotic MIN6 cells was significantly increased in co‐culture with RGN‐KO RAW264.7 macrophages compared with co‐culture with scramble control RAW264.7 macrophages, indicating that RGN deficiency in RAW264.7 macrophages decreased the inhibitory effect of derrisfolin A on co‐culture‐induced MIN6 cell apoptosis. Collectively, these results suggested that in the co‐culture conditions, derrisfolin A inhibited the incidence of LPS‐induced paracrine effects of RAW264.7 macrophages on MIN6 cell apoptosis by inducing RGN expression in RAW264.7 macrophages.

Next, we examined the effect of derrisfolin A on the levels of proinflammatory mediators secreted from LPS‐treated RAW264.7 macrophages into culture medium (Fig. 5B). In the RAW264.7 macrophages alone culture, treatment of scramble control RAW264.7 macrophages with LPS showed a marked increase in the production of NO, TNF‐α, and IL‐1β, and this increase was inhibited by derrisfolin A pretreatment. Additionally, although there was no significant difference in the production of the three proinflammatory mediators between the scramble control and RGN‐KO RAW264.7 macrophages, the RGN‐KO RAW264.7 macrophages showed a decreased inhibitory effect of derrisfolin A on LPS‐induced production of the three proinflammatory mediators. These results suggested that derrisfolin A attenuated the production of the three proinflammatory mediators in RAW264.7 macrophages by inducing RGN expression. Furthermore, we examined the effect of derrisfolin A on the levels of three proinflammatory mediators in culture medium in co‐culture with RAW264.7 macrophages (Fig. 5C). Upon LPS treatment, co‐culture with scramble control RAW264.7 macrophages exhibited a significant increase in the levels of the three proinflammatory mediators in the culture medium in comparison with a culture of MIN6 cells alone, and this increase was inhibited by derrisfolin A pretreatment. Additionally, following LPS treatment, co‐culture with RGN‐KO RAW264.7 macrophages showed no significant increase in levels of the three proinflammatory mediators in the culture medium relative to co‐culture with scramble control RAW264.7 macrophages. Interestingly, following derrisfolin A pretreatment, co‐culture with RGN‐KO RAW264.7 macrophages showed higher levels of the three proinflammatory mediators in the culture medium relative to the co‐culture with scramble control RAW264.7 macrophages, indicating that RGN deficiency in RAW264.7 macrophages attenuated the inhibitory effect of derrisfolin A on co‐culture‐induced increase in levels of the three proinflammatory mediators in the culture medium. Taken together, these results suggested that in the co‐culture condition, derrisfolin A inhibited LPS‐induced production of three proinflammatory mediators in not only MIN6 cells but also RAW264.7 macrophages, resulting in decreased levels of the three proinflammatory mediators in culture medium in co‐culture with RAW264.7 macrophages. Collectively, considering the protective effect of NO scavenger c‐PTIO or a mixture of neutralizing antibodies against IL‐1β and TNF‐α on LPS‐induced MIN6 cell apoptosis in co‐culture conditions (Fig. S2C), our findings suggested that in co‐culture conditions, derrisfolin A suppressed the incidence of LPS‐induced paracrine effects of RAW264.7 macrophages on MIN6 cell apoptosis mediated by the three inflammatory mediators.

We further examined whether derrisfolin A inhibits oxidative/nitrosative stress damage in MIN6 cells co‐cultured with RAW264.7 macrophages in the presence of LPS (Fig. 5D). Following LPS treatment, co‐culture with scramble control RAW264.7 macrophages showed a significant increase in the levels of ROS generation, MAD content, and 3‐NT content in MIN6 cells in comparison with a culture of MIN6 cells alone, and this increased oxidative/nitrosative stress in the MIN6 cells was inhibited by derrisfolin A pretreatment. Additionally, following LPS treatment, co‐culture with RGN‐KO RAW264.7 macrophages exhibited no significant increase in the levels of ROS generation, MAD content, and 3‐NT content in MIN6 cells relative to the co‐culture with scramble control RAW264.7 macrophages. However, after pretreatment with derrisfolin A, the levels of ROS generation, MAD content, and 3‐NT content in MIN6 cells were higher in co‐culture with RGN‐KO RAW264.7 macrophages than scramble control RAW264.7 macrophages. These results suggested that derrisfolin A attenuated the incidence of LPS‐induced paracrine effects of RAW264.7 macrophages on oxidative/nitrosative stress damage in MIN6 cells by inducing RGN expression in RAW264.7 macrophages.

**Fig. 6:**
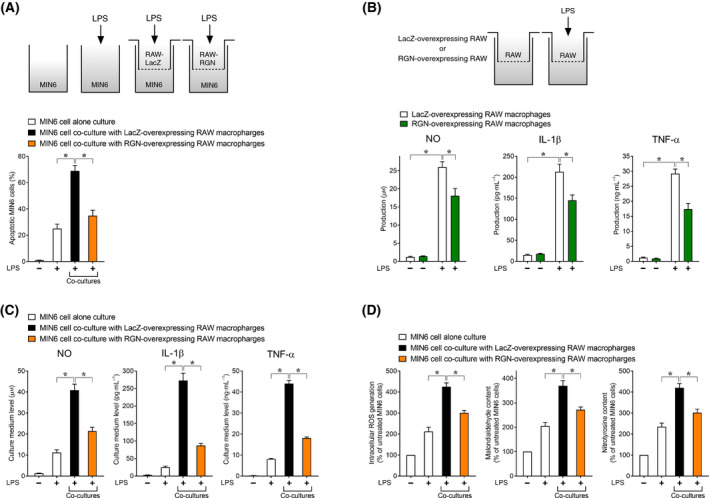
RGN overexpression inhibited the incidence of LPS‐induced paracrine effects of RAW264.7 macrophages on inflammatory cytotoxicity in MIN6 cells under co‐culture conditions. (A) MIN6 cells were seeded in the bottom of a 24‐well plate with or without RAW264.7 macrophages overexpressing LacZ or RGN in the inserts. The cells were treated with or without LPS (100 ng·mL^−1^) for 36 h and then processed to assay the number of apoptotic cells. (B) RAW264.7 macrophages overexpressing LacZ or RGN were treated with or without LPS (100 ng·mL^−1^) for 36 h and then processed to assay the amount of NO, IL‐1β, and TNF‐α in the culture medium. (C and D) MIN6 cells were seeded in the bottom of a 24‐well plate with or without RAW264.7 macrophages overexpressing LacZ or RGN in the inserts. The cells were treated with or without LPS (100 ng·mL^2212^1) for 36 h and then processed to assay the levels of proinflammatory mediators in the culture medium (C) and intracellular ROS generation, malondialdehyde content, and 3‐NT content (D). Each assay was performed as described in the Fig. 3 legend. All data are presented as means ± SE from three independent experiments performed in triplicate. One‐way ANOVA followed by Tukey’s *post hoc* test was performed to compare significance differences among groups. *P < 0.05 was considered statistically significant.

We explored whether RGN induction itself in RAW264.7 macrophages blocked the incidence of LPS‐induced paracrine effects of RAW264.7 macrophages on MIN6 cell damage by employing co‐culture with RGN‐overexpressing RAW264.7 macrophages. Co‐culture with RGN‐overexpressing RAW264.7 macrophages exhibited a significantly lower number of apoptotic MIN6 cells relative to co‐culture with LacZ‐overexpressing RAW264.7 macrophages (Fig. 6A). Additionally, RGN overexpression in RAW264.7 macrophages decreased LPS‐induced production of NO, IL‐1β, and TNF‐α (Fig. 6B) and led to decreased levels of the three proinflammatory mediators in the culture medium in the co‐culture conditions (Fig. 6C). Considering the apoptotic effect of the three proinflammatory mediators on MIN6 cells in co‐culture conditions (Fig. S2C), these results suggested that RGN overexpression in RAW264.7 macrophages blocked the incidence of LPS‐induced paracrine effects of RAW264.7 macrophages on MIN6 cell apoptosis mediated by the three proinflammatory mediators. Furthermore, following LPS treatment, significantly decreased levels of ROS generation, MAD content, and 3‐NT content in MIN6 cells were observed in co‐culture with RGN‐overexpressing RAW264.7 macrophages relative to co‐culture with LacZ‐overexpressing RAW264.7 macrophages (Fig. 6D). Collectively, these results suggested that RGN overexpression in RAW264.7 macrophages blocked the incidence of LPS‐induced paracrine effects of RAW264.7 macrophages on apoptosis and oxidative/nitrosative stress damage in MIN6 cells. The RGN overexpression data described above supported the ability of derrisfolin A‐mediated RGN induction itself to suppress the incidence of LPS‐induced paracrine effect of RAW264.7 macrophages on inflammatory cytotoxicity in MIN6 cells.

## Discussion

Previous studies indicate that T2D in humans is linked to compositional changes in the intestinal microbiota and that Gram‐negative bacteria, which express LPS, are enriched among the gut microbiota in T2D patients [21, 22]. Furthermore, the serum concentration of LPS in diabetic patients has been reported as higher compared with that in healthy controls, indicating LPS as a risk factor for T2D development [23]. LPS, an exogenous bacterial endotoxin, induces an inflammatory response in pancreatic β‐cells by binding to Toll‐like receptors [24, 25, 26]. LPS induces proinflammatory mediators via NF‐κB activation in pancreatic β‐cells [24, 25, 26] and then impair β‐cell function [27], leading to decreased insulin secretion potential. Thus, it is possible that circulating LPS directly attacks β‐cells and triggers β‐cell inflammation and dysfunction. In this study, we found that derrisfolin A‐mediated and gene transfer‐mediated RGN induction attenuated LPS‐induced production of proinflammatory mediators, such as NO, IL‐1β, and TNF‐α, in MIN6 β‐cells by blocking NF‐κB activation. Our findings imply that a chemical inducer of RGN exerts an anti‐inflammatory effect in pancreatic β‐cells. We also found that derrisfolin A‐mediated and gene transfer‐mediated RGN induction attenuated LPS‐induced MIN6 cell apoptosis caused by autocrine toxic effects of the three proinflammatory mediators and then protected MIN6 cells from LPS‐induced oxidative/nitrosative damage. Our findings suggest that derrisfolin A, a chemical inducer of RGN, has potential as a therapeutic agent against inflammation‐mediated β‐cell dysfunction in LPS‐dependent metabolic endotoxemia.

In this study, we found that in both MIN6 cells and RAW264.7 macrophages, RGN induction by derrisfolin A and gene transfer attenuated NF‐κB activation, which was accompanied by the upregulation of inflammatory mediators. It was reported that RGN suppressed oxidative stress‐induced NF‐κB activation caused by a protein tyrosine kinase/protein phosphatase imbalance and protein serine/threonine phosphatase inactivation [41]. RGN induction by derrisfolin A and gene transfer might also attenuate oxidative stress‐induced NF‐κB activation after LPS treatment by modulating the protein tyrosine kinase/protein phosphatase balance.

It was reported that macrophages infiltrate pancreatic islets in T2D patients and model animals, such as Goto‐Kakizaki rats, *db*/*db* mice, and high‐fat d‐fed mice [28, 42, 43]. Communication between macrophages and β‐cells in the inflammatory islet microenvironment is mediated by proinflammatory mediators. The resident islet macrophages secrete proinflammatory cytokines, such as IL‐1β and TNF‐α, leading to β‐cell dysfunction within the islets [29, 30, 31, 32]. In this study, we found that derrisfolin A‐mediated and gene transfer‐mediated RGN induction in RAW264.7 macrophages suppressed LPS‐induced upregulation of proinflammatory mediators, such as NO, IL‐1β, and TNF‐α, by blocking NF‐κB activation. Furthermore, we simulated the inflammatory microenvironment in T2D islets by co‐culturing MIN6 cells and RAW264.7 macrophages in the presence of LPS and demonstrated that in co‐culture conditions, derrisfolin A‐mediated and gene transfer‐mediated RGN induction in RAW264.7 macrophages attenuated the incidence of LPS‐induced paracrine effects of RAW264.7 macrophages on apoptosis and oxidative/nitrosative stress in MIN6 cells, accompanied by decreased levels of NO, IL‐1β, and TNF‐α in the co‐culture medium. Our present data warrant the consideration of RGN as a therapeutic target for macrophage‐mediated β‐cell inflammation in T2D. Thus, derrisfolin A, a chemical inducer of RGN, may act as a potential anti‐inflammation compound targeting islet‐associated inflammatory macrophages leading to β‐cell death.

In both MIN6 cells and RAW264.7 macrophages, following LPS treatment, derrisfolin A pretreatment exhibited the lower mRNA expression and production of inflammatory mediators compared with gene transfer‐mediated RGN induction (see derrisfolin A effect in scramble cells in Fig. 1D versus RGN overexpression effect in Fig. 2A; derrisfolin A effect in scramble cells in Fig. 3B versus RGN overexpression effect in Fig. 4B; derrisfolin A effect in scramble cells in Fig. 5B versus RGN overexpression effect in Fig. 6B). These results imply that a mechanism other than RGN induction also contributes to the anti‐inflammatory effects of derrisfolin A. Microarray analysis revealed six genes associated with stress responses and anti‐apoptosis including RGN, whose expressions were 10‐fold above changed by derrisfolin A (unpublished data). It is possible that the change in the expression of derrisfolin A‐responsive genes also contributes to the anti‐inflammatory effect of this compound. Further studies should elucidate the detailed mechanisms of derrisfolin A‐induced anti‐inflammatory activity.

Considering the beneficial effect of derrisfolin A on inflammatory responses in both MIN6 cells and RAW264.7 macrophages, the induction of RGN expression in both β‐cells and islet‐associated macrophages may be a novel therapeutic strategy targeted to both the preservation of β‐cell survival and the inhibition of macrophage activation in islet inflammation caused by T2D. Therefore, pharmacological *in vivo* experiments using derrisfolin A are required to examine the efficacy of this compound in blocking inflammatory interaction between β‐cells and macrophages in the islets of T2D model animals. Further *in vivo* investigations of the anti‐inflammatory effects of RGN on β‐cell inflammation could lead to a better understanding of the pathogenic mechanism of macrophage‐mediated β‐cell dysfunction in T2D model animals.

## Conflict of interest

The authors declare no conflict of interest.

## Author contributions

TM, K. Hashimoto, CI, MI, RK, and K. Hikita performed the experiments; SK and CT constructed the retrovirus expression system; and TM and MY designed the experiment and discussed it with NK. MY provided comments pertaining to the manuscript, and TM and K. Hashimoto wrote the manuscript. All authors read and commented on the manuscript.

## Supporting information


**Fig. S1.** Structure of derrisfolin A isolated from the stems of *Derris trifoliata* Lour. (Leguminosae).
**Fig. S2.** Inflammatory mediators trigger apoptosis in MIN6 cells treated with LPS and co‐cultured with RAW264.7 macrophages in the presence of LPS. (A and B) MIN6 cells were seeded in the bottom of a 24‐well plate with or without RAW264.7 macrophages in the inserts and then treated with or without LPS (100 ng/mL) for 36 h. Thereafter, TUNEL‐positive MIN6 apoptotic cells were assayed (A), and the levels of proinflammatory mediators in the culture medium (B) were determined, as described in the Fig. 3 legend. (C) MIN6 cells were co‐cultured with or without RAW264.7 cells in the presence of LPS (100 ng/mL) for 36 h, along with either NO scavenger, c‐PTIO (25 μM), a mixture of anti‐IL‐1β and anti‐TNF‐α neutralizing antibodies (100 ng/ml), or isotype control IgG. Thereafter, the number of TUNEL‐positive apoptotic cells was determined. All data are presented as means ± SE from three independent experiments performed in triplicate. One‐way ANOVA followed by Tukey’s post hoc test was performed to compare significance differences among groups. *P < 0.05 was considered statistically significant.

## Data Availability

The data that support the findings of this study are provided in the figures of this article.
