# Supplementary figures and images for: Chemical inducer of regucalcin attenuates lipopolysaccharide‐induced inflammatory responses in pancreatic MIN6 β‐cells and RAW264.7 macrophages
Source: FEBS Open Bio. 2021 Nov 9;12(1):175–91. doi: 10.1002/2211-5463.13321 (PMC8727933; doi:10.1002/2211-5463.13321)

Figure S1

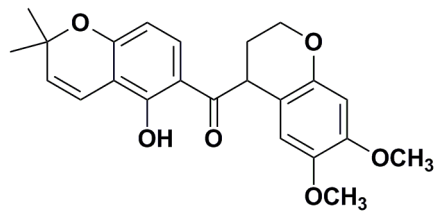

Figure S2

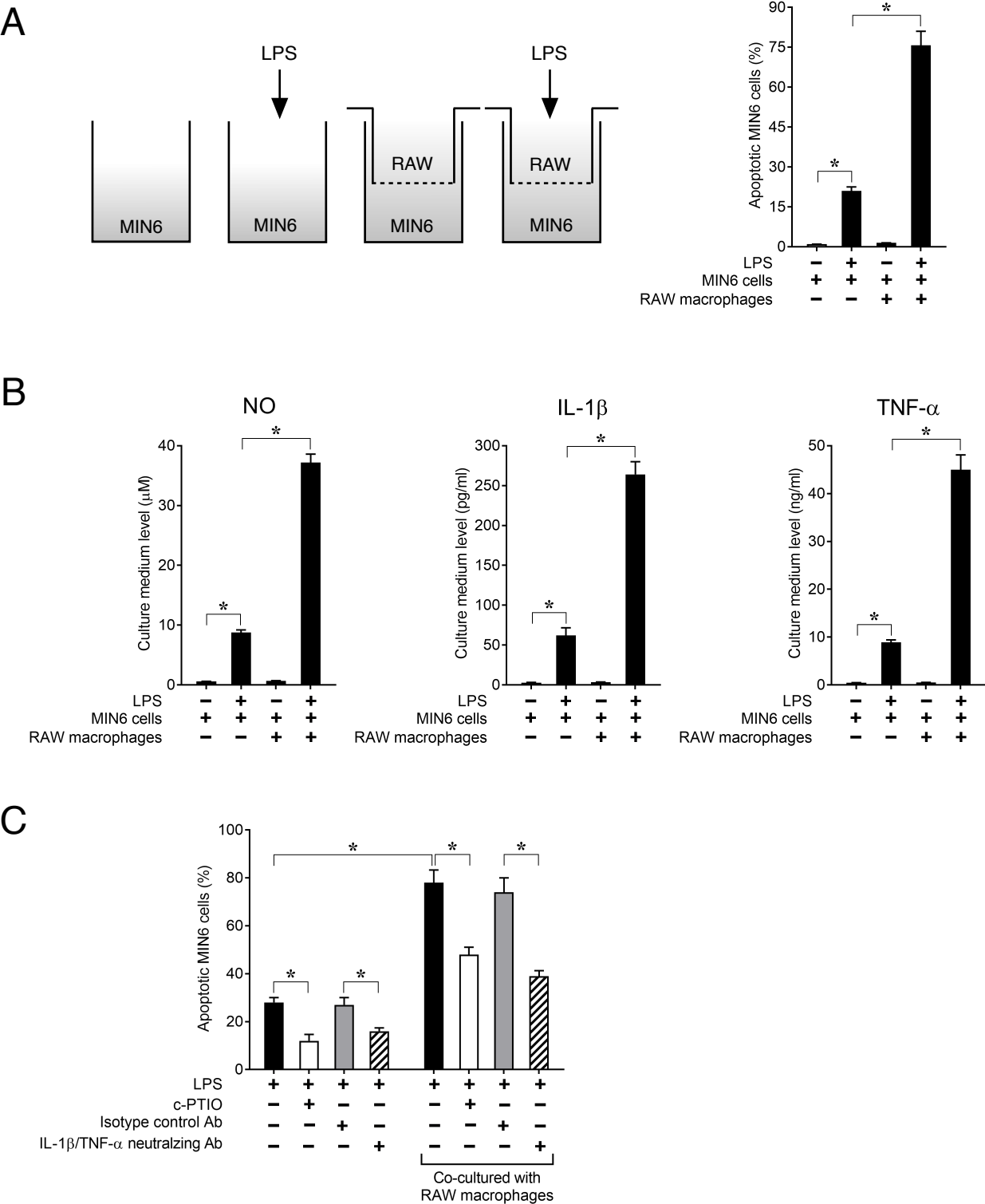

Supplement: Supplementary file 1 — Fig. S1. Structure of derrisfolin A isolated from the stems of Derris trifoliata Lour. (Leguminosae). Fig. S2. Inflammatory mediators trigger apoptosis in MIN6 cells treated with LPS and co‐cultured with RAW264.7 macrophages in the presence of LPS. (A and B) MIN6 cells were seeded in the bottom of a 24‐well plate with or without RAW264.7 macrophages in the inserts and then treated with or without LPS (100 ng/mL) for 36 h. Thereafter, TUNEL‐positive MIN6 apoptotic cells were assayed (A), and the levels of proinflammatory mediators in the culture medium (B) were determined, as described in the Fig. 3 legend. (C) MIN6 cells were co‐cultured with or without RAW264.7 cells in the presence of LPS (100 ng/mL) for 36 h, along with either NO scavenger, c‐PTIO (25 μM), a mixture of anti‐IL‐1β and anti‐TNF‐α neutralizing antibodies (100 ng/ml), or isotype control IgG. Thereafter, the number of TUNEL‐positive apoptotic cells was determined. All data are presented as means ± SE from three independent experiments performed in triplicate. One‐way ANOVA followed by Tukey’s post hoc test was performed to compare significance differences among groups. *P < 0.05 was considered statistically significant. [file FEB4-12-175-s001.pdf]
